# The Story of the Insane from Year to Year

**Published:** 1902-12-20

**Authors:** 


### THE STORY OF THE INSANE FROM YEAR TO YEAR.

FIFTEENTH ANNUAL SERIES.

### WEST RIDING ASYLUM AT WAKEFIELD.

ON January 1st this asylum contained 1,469 patients, and on December 31st the number was 1,658. There were 566 first admissions, 91 readmissions, 238 discharged recovered, 58 relieved, and 170 deaths. The average number resident was 1,589, and the total number under care during the year was 2,105. The recoveries stand at 36·2 per cent. of the admissions, but if the transfers be excluded the percentage rises to 41·82 per cent. The deaths are 10·59 per cent. of the average number resident. Of the deaths 61 were caused by cerebral and spinal diseases, 20 by consumption, 14 by other lung diseases, 8 by enteritis, and 2 by enteric fever. Of the year's admissions it is stated that domestic trouble was the cause of insanity in 46, mental anxiety in the same number, religion in 32, intemperance in drink in 119, hereditary influence in 170, and previous attacks in 141. Dr. Lewis' report runs to over twenty pages and contains many interesting paragraphs. When speaking of drink as a cause of insanity he tells us that for the past five years it was the cause in over 23 per cent. of the cases admitted to his asylum. Many years since all forms of alcohol were omitted from the ordinary dietary, and we notice that of 328 cases admitted to the acute hospital it was only prescribed in three instances. The committee "record their high estimation" of the management of the asylum by Dr. Bevan Lewis. The weekly cost of maintenance is 11s. 2d. per head.

### GLAMORGAN COUNTY ASYLUM AT BRIDGEND.

The number of patients in this asylum was, on January 1st, 1,658, and on December 31st, 1,841. There were 486 first admissions, 74 readmissions, 144 discharged recovered, 32 discharged relieved, and 167 deaths. The average number resident was 1,744, and the total number under treatment was 2,207. The recoveries were 26·2 per cent. on the admissions, and the deaths were 7·5 per cent. on the average number resident, both of these percentages being below the county asylum averages, and also below the Bridgend average for many years. Of the year's deaths 71 were owing to cerebral and spinal diseases (general paralysis accounting for 45 of these), 26 were caused by consumption, and 13 by other lung diseases. Table X. shows that 107 of the cases admitted owed their insanity to moral causes, domestic trouble being the chief factor, intemperance in drink accounts for 143, and hereditary influence for 148. A temporary ward for 150 men was opened in the spring, but in spite of this the asylum contained 140 more patients on the date of the report than its proper complement. It is proposed to build a temporary ward for 100 women, and 40 patients were transferred to other asylums. When speaking of the causes of insanity, Dr. Pringle very truly says that in many cases no cause could be traced because the "friends enter into a conspiracy of silence or direct denial from false ideas of loyalty and honour;" but, he adds, "that about one-fourth of all the cases admitted should be due to a preventable cause—intemperance in drink—is, I think, a most grave blot upon our age and so-called civilisation." The committee refer "to the rare administrative ability and uniform kindness exhibited by the medical superintendent." The average weekly cost per head is 9s. 3d.

### LONDON COUNTY ASYLUM AT CLAYBURY.

The number of patients resident on January 1st was 2,505, and on the last day of the year the number had fallen to 2,406. There were 364 first admissions, 62 readmissions, 148 discharged recovered, 176 discharged relieved, and 201 died. The average number resident during the year was 2,431. Calculated on the admissions the recoveries stand at 37·95 per cent., and calculated on the average number resident the deaths stand at 8·27 per cent. Of the year's deaths 69 were caused by cerebral and spinal diseases general paralysis being responsible for 50 of that number, 18 by consumption, 22 by other lung diseases, and 21 by colitis. Of the year's admissions 51 were supposed to owe their insanity to such moral causes as domestic trouble, adverse circumstances, and religion. Among the physical causes, previous attacks head the list with 78 cases, heredity comes next with 50 cases, and drink follows closely with 48. When speaking of the admissions Dr. Jones says "that the two classes which furnish the greatest number of male admissions are described as "clerks" and "persons of no occupation." Given a normal heredity, the proper environment for mental health is one that can supply a constant stream of ingoing sensorial impressions to the cerebral cortex, and a deprivation of these means mental starvation and disease. Undoubtedly, idleness is the mother of much more than mischief, and the man who is prone to nervous disease of any kind should carefully guard against its Satanic influence. Asylum dysentery, or colitis, attacked 121 patients, and was the cause of death, as already stated, in 21 cases. With such an extensive experience of this curious disease, it would have been both interesting and instructive to have Dr. Jones' views on its causation and prevention. He names three conditions as probably influential—bad drainage, overcrowding, and non-isolation of cases of diarrhœa; and now all these conditions have been done away with at Claybury. We do not wish to disturb Dr. Jones' equanimity; but we much fear the disease will assert its superiority over good drainage, cubic space, and isolation, although the alterations are good things to have accomplished, and no doubt will tend to diminish the number of cases stricken with this asylum scourge. Phthisis pulmonalis accounts for 18 deaths and other forms of tuberculosis for 6, making a total of 24, and being nearly 12 per cent. of the whole number of deaths. This percentage of deaths, although lower than in many asylums, must be representative of a very considerable number of cases of consumption. As the Claybury Asylum is marching in the van of the metropolitan asylums, we venture to ask whether it intends being first to isolate its tuberculous patients, and whether Dr. Jones has formulated any scheme whereby this isolation could be carried out with success and reasonable economy. The visitors say that "Dr. Jones and the asylum staff generally have been assiduous in the performance of their responsible duties.' The weekly rate of maintenance was nearly 12s. 3d. per head.

### THREE COUNTIES' ASYLUM AT ARLSEY, HERTS.

This asylum contained 925 patients on the first day of January, and by the end of December the number had increased to 999. The average number resident during the year was 952, and the total number under treatment was 1,215. There were 177 first admissions, 34 readmissions, 84 discharged recovered, 7 discharged relieved, and 106 died The recoveries were 28·9 per cent. of the admissions, and the deaths were 11·1 per cent. of the average number resident. The recoveries are less numerous than the County Asylum average, and are also about 5 per cent. lower than the Three Counties' average for 40 years. But if transfers be excluded the recovery rate rises to 39·3 per cent., which is very fair. The deaths are higher than the average. Of these deaths 26 were caused by cerebral and spinal diseases, 19 by consumption, and 8 by other lung diseases. Of the year's admissions moral causes account for the insanity in 45 instances, and of the physical causes hereditary influences account for 51, previous attacks for 34, and intemperance in drink for 17. There was no death from any infectious disease, and asylum dysentery did not make its appearance, for which absence Dr. de Lisle is to be congratulated. One of the patients had obtained and sharpened a piece of steel from a corset and therewith attacked one of the assistant medical officers. Very properly he was handed over to the police, was tried at the Assizes, and sentenced to be detained at Broadmoor Criminal Asylum during His Majesty's pleasure. Such attacks are by no means infrequent in our county asylums, and we believe they would be less frequent if the example of the Three Counties' Asylum were more generally followed. No Coroner's inquest had to be held during the year, and neither restraint nor seclusion had to be employed. The Visiting Committee say that "the management of the asylum has been carried out in a most efficient manner in all respects by Dr. de Lisle." The weekly cost of maintenance was 9s. 4½d. per head.

### WESTERN COUNTIES' ASYLUM FOR IDIOTS, AT STARCROSS, DEVON.

HERE the numbers were stationary during the year, there having been 269 in residence on January 1st, and the same number on December 31st. There were 46 admissions, 3 discharged recovered, 16 discharged much improved, 14 moderately improved, 2 not improved, and 11 died. Of the children admitted 23 were under 10 years of age, 16 were between 10 and 15, and 7 above the latter age. In a great many cases the training of idiot children is an almost hopeless task, and it is invariably uphill work. The figures which we have quoted above will show that a very considerable degree of success has followed on the labour of the officials at Starcross. On discharge not fewer than 10 were in a position to earn their own living. Of course many of those discharged, although they could not go so far as to earn a livelihood, or to stand alone, as the superintendent expresses it, were still capable of helping in the work of their homes. It is beyond doubt that but for the training received they would have gone from bad to worse, and would have ended in the imbecile wards of a workhouse or an asylum, being a burden on the rates as long as they lived. Hence, without emphasising the Christian side of the question, these idiot asylums deserve the support of the charitable from the economical side merely. All the usual trades are carried on. Thirty-three of the boys learn shoemaking, 41 tailoring, 44 basket-making, 21 mat-making, and others are employed at carpentering, carving, and gardening. The girls find useful occupation in the laundry, the kitchen, and the sewing-room. The income for the year was £10,079, and £7,700 was obtained from payments received for the maintenance of patients. The weekly cost per head was 8s. 8d.

### ROYAL ASYLUM, MONTROSE.

HERE 675 patients were in residence at the beginning of the year and 679 at the end of it. There were 152 first admissions, 37 readmissions, 74 discharged recovered, 16 discharged relieved, and 95 died. The average number resident was 690, and the total number under care was 862. The recoveries stand at 39·15 per cent. of the admissions, and the deaths at 13·76 on the average number resident. The latter is much above the average at the Montrose Asylum, and Dr. Havelock attributes it to an outbreak of influenza which carried off a number of feeble, senile cases. Of the year's deaths, 32 were caused by cerebral and spinal diseases, 7 by consumption, 14 by pneumonia, and 1 by general tuberculosis. Of the year's admissions the insanity was due to moral causes in 11 instances, while of the physical causes, intemperance in drink accounts for 16, hereditary influence for 34, and in 68 no cause is stated. Dr. Havelock is not a convert to the belief that insanity is increasing among us. "There is no doubt," he says, "that the number of those certified as insane and fit subjects for asylum care is annually increasing; but it is by no means proved that there is an actual increase of insanity relative to the general population." We have often quoted the opinions of medical superintendents on the opposite side of the question, and we gladly print the above extract; but at the same time we think, although it would be difficult to prove it, that making every possible allowance for the accumulation of demented cases in asylums, there is some increase of insanity in Great Britain. The asylum contains about 120 private patients, and five voluntary boarders.

### LONDON COUNTY ASYLUM AT COLNEY HATCH.

HERE 2,538 patients were in residence at the beginning of January, and on December 31st the number had fallen to 2,500. There were 389 first admissions, 84 readmissions, 194 discharged recovered, 111 discharged relieved, and 206 died. The average number resident was 2,505. Excluding transfers, the recoveries stand at 43·40 per cent. of the admissions, and the deaths at 8·22 per cent. of the average number resident. The recovery rate is high and the death rate is rather under the average. Of the year's deaths, 87 were caused by cerebral and spinal diseases, 29 by consumption, 12 by other lung-diseases, 29 by colitis and enteritis, and 5 by enteric fever. Of the year's admissions, the insanity is stated to have resulted from various moral causes—domestic trouble, religion, shock, etc., in 69 instances, and among the physical causes, intemperance in drink accounts for 61 cases, hereditary influence for 118, and previous attacks for 134. In 127 the cause was unknown. The statistics of this asylum throw a curious side-light on the evils of alien immigration, as no fewer than 71 of the year's admissions were Jews, the great majority being Russian Poles. The expenses attending these will not amount to less than £1,850 a year, and these expenses will grow annually. When speaking of the deaths, Dr. Seward states that no serious sanitary defects could be detected to account for the enteric fever and the colitis. Theformer attacked four nurses and 13 patients, and the latter 60 patients. This peculiarity of colitis in selecting patients and passing by the staff has been again and again noticed in asylums, while enteric fever shows no such preference. If anything, a higher proportion of the attendants and nurses is attacked by it. The committee express their "appreciation of the thoroughly efficient manner in which Dr. Seward and the medical and general staff have performed their responsible duties."

